# Integrated Bioinformatics-Based Analysis of Hub Genes and the Mechanism of Immune Infiltration Associated With Acute Myocardial Infarction

**DOI:** 10.3389/fcvm.2022.831605

**Published:** 2022-04-06

**Authors:** Yanze Wu, Ting Jiang, Jinghai Hua, Zhiping Xiong, Hui Chen, Lei Li, Jingtian Peng, Wenjun Xiong

**Affiliations:** ^1^Department of Cardiology, The First Affiliated Hospital of Nanchang University, Nanchang, China; ^2^Jiangxi Medical College, Nanchang University, Nanchang, China; ^3^Department Hospital Infection Control, The First Affiliated Hospital of Nanchang University, Nanchang, China

**Keywords:** acute myocardial infarction, immune cell infiltration, hub gene, bioinformatics, CIBERSORT

## Abstract

**Background:**

Acute myocardial infarction (AMI) is a fatal disease that causes high morbidity and mortality. It has been reported that AMI is associated with immune cell infiltration. Now, we aimed to identify the potential diagnostic biomarkers of AMI and uncover the immune cell infiltration profile of AMI.

**Methods:**

From the Gene Expression Omnibus (GEO) data set, three data sets (GSE48060, GSE60993, and GSE66360) were downloaded. Differentially expressed genes (DEGs) from AMI and healthy control samples were screened. Furthermore, DEGs were performed *via* gene ontology (GO) functional and kyoto encyclopedia of genes and genome (KEGG) pathway analyses. The Gene set enrichment analysis (GSEA) was used to analyze GO terms and KEGG pathways. Utilizing the Search Tool for Retrieval of Interacting Genes/Proteins (STRING) database, a protein–protein interaction (PPI) network was constructed, and the hub genes were identified. Then, the receiver operating characteristic (ROC) curves were constructed to analyze the diagnostic value of hub genes. And, the diagnostic value of hub genes was further validated in an independent data set GSE61144. Finally, CIBERSORT was used to represent the compositional patterns of the 22 types of immune cell fractions in AMI.

**Results:**

A total of 71 DEGs were identified. These DEGs were mainly enriched in immune response and immune-related pathways. Toll-like receptor 2 (TLR2), interleukin-1B (IL1B), leukocyte immunoglobulin-like receptor subfamily B2 (LILRB2), Fc fragment of IgE receptor Ig (FCER1G), formyl peptide receptor 1 (FPR1), and matrix metalloproteinase 9 (MMP9) were identified as diagnostic markers with the value of *p* < 0.05. Also, the immune cell infiltration analysis indicated that TLR2, IL1B, LILRB2, FCER1G, FPR1, and MMP9 were correlated with neutrophils, monocytes, resting natural killer (NK) cells, gamma delta T cells, and CD4 memory resting T cells. The fractions of monocytes and neutrophils were significantly higher in AMI tissues than in control tissues.

**Conclusion:**

TLR2, IL1B, LILRB2, FCER1G, FPR1, and MMP9 are involved in the process of AMI, which can be used as molecular biomarkers for the screening and diagnosis of AMI. In addition, the immune system plays a vital role in the occurrence and progression of AMI.

## Introduction

Acute myocardial infarction (AMI) is one of the leading causes of adverse outcomes in developed countries (1). Despite improved pharmacologic and device-based approaches to ameliorate AMI, no significant improvement in morbidity and mortality has been observed over the past 20 years (2). Currently, the diagnosis of AMI is often based on changes in cardiac biomarkers. Numerous biomarkers have been used for the clinical diagnosis of AMI, such as cardiac troponin I, cardiac troponin T, and MB isoenzyme of creatine kinase (CK-MB) (3, 4). However, there are limitations in sensitivity and specificity for the early diagnosis of AMI through these biomarkers (5). In addition, the classic risk factors for AMI, such as smoking, obesity, hypertension, and high serum cholesterol, can only predict the prevention and outcomes of AMI and fail to provide an acute diagnosis sufficiently (6).

Fortunately, with the development of gene chip technique and transcriptome sequencing methods, more and more gene chip technologies are used in cardiovascular clinics and studies, which contribute to the identification of novel biomarkers for the early diagnosis and prognosis of diseases. Zhang et al. found that ARG1 might play a key role in the development of AMI, which could be a biomarker of AMI and provide a reference for intensive research (7). Chen et al. identified TBX21 and PRF1 as novel diagnostic biomarkers and as potential modulatory targets *via* analyzing the microarray expression profile of peripheral patients with AMI (8). Furthermore, research has shown that a variety of immune cells play an increasingly significant role in the immunomodulation after AMI (9). M2 macrophages, mast cells, and eosinophils have been proven to participate in affecting cardiac after AMI, which provide new insights into the immune mechanisms in the development of AMI (10).

In this study, three microarray data sets were downloaded from GEO. They were merged into a metadata cohort and used to screen out differentially expressed genes (DEGs) between AMI and controls. Next, gene ontology (GO) functional and Kyoto encyclopedia of genes and genome (KEGG) pathway analyses were performed based on DEGs. Moreover, the gene set enrichment analysis (GSEA) was used to analyze GO terms and KEGG pathways. A protein–protein interaction (PPI) network was constructed using the Search Tool for Retrieval of Interacting Genes/Proteins (STRING) database, and then the hub genes were identified. The receiver operating characteristic (ROC) curves were analyzed and depicted for the diagnostic effectiveness of hub genes. Finally, CIBERSORT was utilized to quantify the composition of immune cells between AMI and normal tissues. Furthermore, we investigate the relationship between the hub genes and infiltrated immune cells for further research.

## Materials and Methods

### Gene Expression Profile Data Collection

The Gene Expression Omnibus (GEO) database collects and shares publicly a range of different high-throughput sequencing and microarray-based data sets. In our research, we searched the data sets, which consisted of patients with AMI and healthy controls. The microarray expression data sets (GSE48060, GSE60993, and GSE66360) were obtained and downloaded from the GEO database^1^, including GSE48060 contributed by Suresh et al., GSE66360 contributed by Kramer et al., and GSE60993 contributed by Eun et al. (11–13). These data sets were based on GPL570 platform of Affymetrix Human Genome U133 Plus 2.0 array and GPL6884 platform of Illumina HumanWG-6 v3.0 expression beadchip, respectively. These three data sets were merged into a metadata, which was used as the training group. Moreover, GSE61144 contributed by Eun et al. was served as an external validation data set (13). The detailed information about our data sets and the annotation platform is listed in Table 1.

**TABLE 1:** Details of the Gene Expression Omnibus (GEO) data sets.

	AMI sample	Control sample	Platforms
GSE60993	7	7	Affymetrix Human Genome U133 Plus 2.0 Array
GSE66360	49	50	Illumina HumanWG-6 v3.0 expression beadchip
GSE48060	31	21	Affymetrix Human Genome U133 Plus 2.0 Array
GSE61144	7	10	Sentrix Human-6 v2 Expression BeadChip

### Identification of DEGs

Prior to the DEG analysis, the preprocessing step was performed. If a gene had more than one probe site, we averaged the values of the probe sites. The probe IDs were converted to the gene symbol based on the annotation files from the platform, and the probes that did not correspond to the genes symbol were excluded.

Furthermore, the three data sets were merged into a metadata cohort and the batch effects were preprocessed and adjusted by the ComBat function of the SVA package (14). In addition, the “limma” of R software was used to perform background correction and array normalization. Meanwhile, DEGs between patients with AMI and healthy controls were identified using the “limma” package (15). The threshold of DEGs in the data set was set as the adjusted value of *p* < 0.05 and | log_2_FC| > 1 (FC: fold change).

### Functional Enrichment Analysis of DEGs

The “Bioconductor” and “GOplot” package of R software were utilized for analyzing the significant DEGs, and to conduct the GO enrichment, which includes molecular functions (MFs), biological processes (BPs), and cellular components (CCs) and KEGG pathway enrichment analyses (16–18). False discovery rate (FDR) <0.05 was regarded as the significantly enriched gene set.

### Gene Set Enrichment Analysis

Using the GSEA software, the enrichment analysis of GO terms, KEGG pathways, and immunologic signature gene sets were performed. We set the number of permutations to 1,000, and set the permutation type as “phenotype.” The reference gene sets used during this analysis were downloaded from the Molecular Signature Database (MSigDB) (19).

### Construction of a PPI Network and the Identification of Hub Genes

To further identify directly or indirectly interacting proteins related to DEGs, the STRING version 11.5 was used to construct a PPI network (20). Meanwhile, we set the confidence score as >0.700 used for network construction. Cytoscape 3.8.2 was utilized for visualizing the network, and the plug-in cyto-Hubba of the Cytoscape software was used to identify the hub genes.

### Validation of Diagnostic Values of Hub Genes

We constructed the ROC curves using the mRNA expression data from 87 AMI and 78 controls *via* SPSS 22.0, and calculated the area under the curve (AUC) of the hub genes, which indicated the diagnostic efficiency of genes. The higher the values of AUC, the greater the diagnostic efficiency of genes. And, the results were further validated in the data set GSE61144. The value of *p* < 0.05 was regarded as statistically significant.

### CIBERSORT

A bioinformatics algorithm called CIBERSORT was used to evaluate immune cell infiltrations. The leukocyte gene signature matrix LM22 with 1,000 permutations was used to calculate the putative abundance of immune cells (21). The data with a CIBERSORT value of *p* < 0.05 were filtered and retained for the following analysis. Thus, a matrix of immune cells fractions was generated. We used the R package “corrplot” to visualize a correlation analysis and 22 types of infiltrated immune cells. The visualization of the differences in immune cell infiltration between AMI and control samples was performed using the R package “vioplot.”

### A Correlation Analysis Between Hub Genes and Immune Cells

We conducted Spearman’s rank correlation analysis of the hub genes and immune cells to further explore the immune mechanism during the development of AMI using the R software. The results were visualized with the “ggplot2” package.

## Results

### Identification of DEG in AMI

**FIGURE 1:**
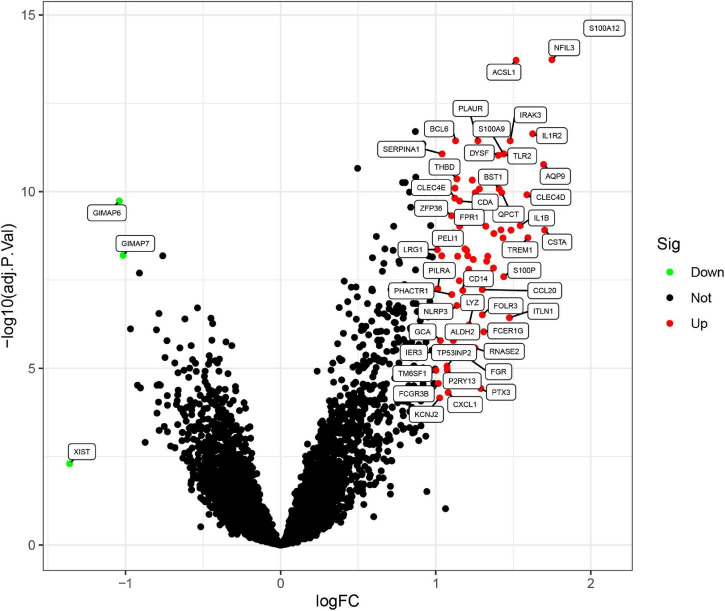
Differentially expressed genes (DEGs) between acute myocardial infarction samples and control ones. The red dots represent upregulated differential genes, the green dots represent differential downregulated genes, and the black dots represent genes without significant differences.

**FIGURE 2:**
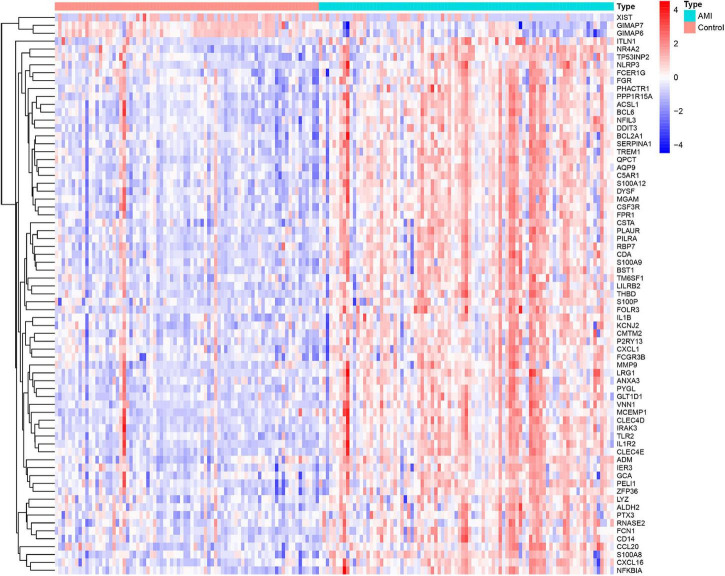
Heatmap showing the expression changes in acute myocardial infarction and control samples. Red represents upregulated DEGs, blue represents downregulated DEGS, and the gradation of color represents the value of | log FC| (FC: fold change).

Three GEO data sets (GSE60993, GSE66360, and GSE48060) were enrolled in this study, and the merged data set included 87 patients with AMI and 78 control samples. After removing the batch effects, the analysis of DEGs was performed using the “limma” package in R software. A total of 71 DEGs include 68 upregulated genes and 3 downregulated genes. The upregulated genes and downregulated genes were significantly separated in the volcano plot (Figure 1). The expression levels of DEGs were presented in the heatmap (Figure 2).

### Functional Enrichment Analysis of DEGs

**FIGURE 3:**
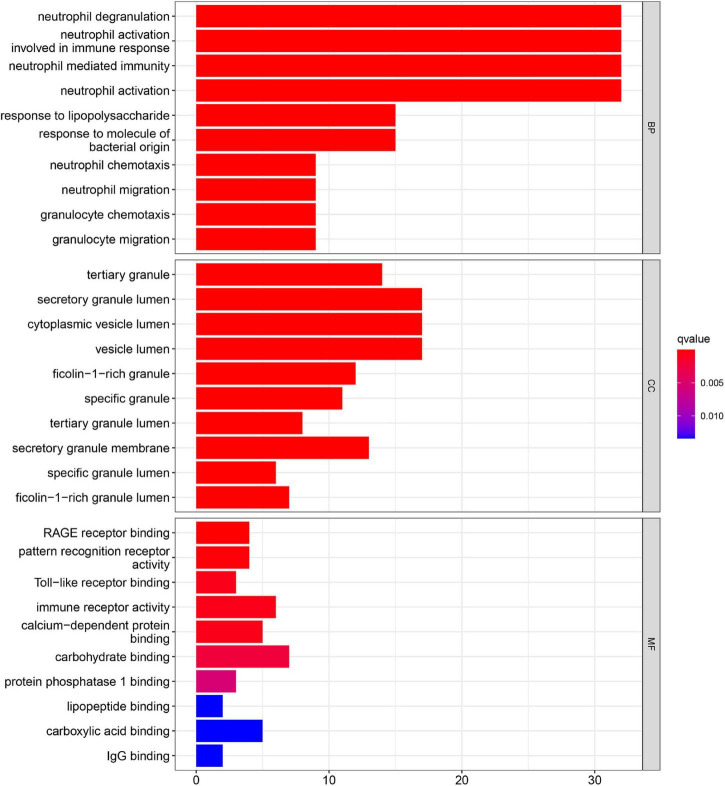
Gene ontology (GO) enrichment analyses of DEGs in acute myocardial infarction (AMI).

Gene ontology function and KEGG pathways enrichment analyses were conducted to gain a deeper insight into the function of DEGs. Figure 3 showed the top 10 enriched GO terms. The GO terms containing three parts: BP, CC, and MF. We mainly focused on BP terms during this study. DEGs of BP were mainly enriched in immune responses (e.g., neutrophil activation, neutrophil-mediated immunity, neutrophil migration, and involvement in immune responses).

**FIGURE 4:**
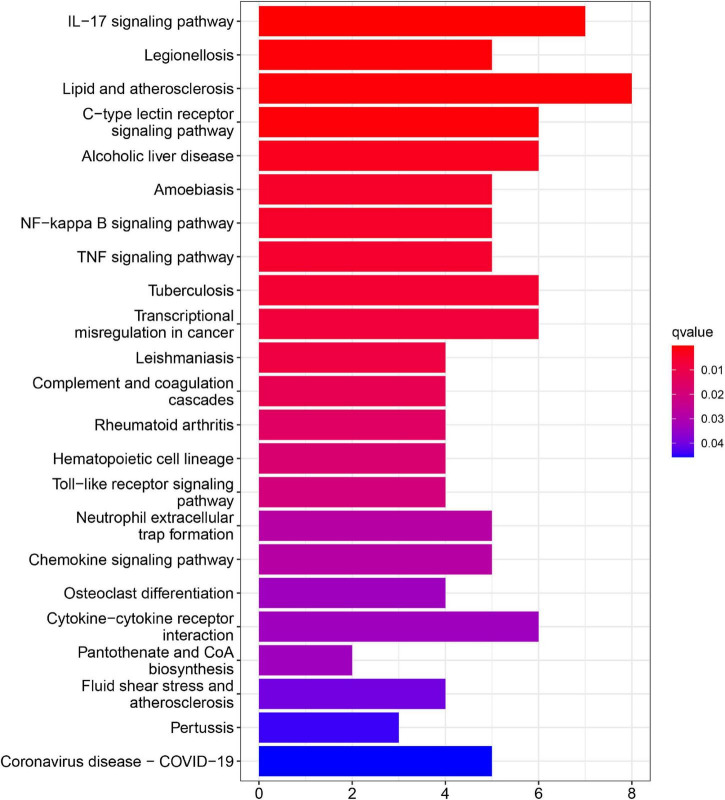
Kyoto encyclopedia of genes and genome (KEGG) pathway enrichment analysis of DEGs in AMI.

In addition, KEGG pathway analyses of DEGs are presented in Figure 4. The results shows the enriched pathways, primarily including immune system diseases (Legionellosis, Amoebiasis, and TB), immune-related pathways [IL-17 signaling pathways, C-type lectin receptor signaling pathway, nuclear factor-kappa B (NF-kappa B) signaling pathway, and tumor necrosis factor (TNF) signaling pathway], and transcriptional misregulation in cancer.

### Gene Set Enrichment Analysis

To explore the potential pathways and gene function associated with the immune system, GO and KEGG analyses were performed using GSEA. The GSEA results are similar to those of the GO and KEGG analyses based on DEGs (Tables 2, 3).

**TABLE 2:** Top 20 significant GO terms enriched by differentially expressed genes (DEGs) *via* gene set enrichment analysis (GSEA), *p* < 0.05.

GO terms	ES	NES	NOM *p*-value
Iron ion transport	0.58	2.33	0.000
Cellular response to external stimulus	0.53	2.17	0.000
P38MAPK cascade	0.72	2.15	0.000
Interleuin-1 production	0.66	2.12	0.000
Negative regulation of NF-kappa B transcription factor activity	0.58	2.11	0.000
Acute phase response	0.78	2.11	0.000
Phagocytosis	0.57	2.09	0.000
Animal organ regeneration	0.60	2.09	0.000
Cellular response to mechanical stimulus	0.59	2.09	0.000
Interleuin-1 beta production	0.67	2.08	0.000
Cellular response to extracellular stimulus	0.50	2.08	0.000
Acute inflammatory response	0.66	2.07	0.000
Positive regulation of interleukin-1 production	0.71	2.07	0.000
Dendritic cell differentiation	0.63	2.05	0.000
Vacuolar lumen	0.56	2.05	0.000
Production of molecular mediator involved in inflammatory response	0.66	2.04	0.000
Transferrin transport	0.58	2.04	0.000
Vascular endothelial growth factor receptor signaling pathway	0.53	2.04	0.000
Smooth muscle cell proliferation	0.60	2.04	0.000
Phagocytic vesicle membrane	0.62	2.04	0.000

*GO, gene otology; ES, enrichment score; NES, normalized enrichment score; NOM p-value, normalized p-value.*

**TABLE 3:** Top 20 significant KEGG pathways enriched by DEGs *via* GSEA, *p* < 0.05.

KEGG terms	ES	NES	NOM *p*-value
Epithelial cell signaling in Helicobacter pylori infection	0.58	2.06	0.000
Fc gamma R-mediated phagocytosis	0.56	2.05	0.000
Leukocyte transendothelial migration	0.49	1.97	0.000
Nod-like receptor signaling pathway	0.64	1.90	0.002
Toll-like receptor signaling pathway	0.59	1.88	0.004
Leishmania infection	0.69	1.88	0.002
MAPK signaling pathway	0.39	1.87	0.000
Adipocytokine signaling pathway	0.47	1.84	0.000
Hematopoietic cell lineage	0.55	1.81	0.000
Pathogenic Escherichia coli infection	0.52	1.80	0.008
Fc epsilon RI signaling pathway	0.46	1.79	0.016
Chemokine signaling pathway	0.43	1.75	0.002
Type II diabetes mellitus	0.49	1.74	0.000
ErbB signaling pathway	0.41	1.71	0.012
Bladder cancer	0.54	1.70	0.004
Complement and coagulation cascades	0.59	1.69	0.002
Galactose metabolism	0.53	1.68	0.017
Pathways in cancer	0.34	1.67	0.000
Renal cell carcinoma	0.41	1.67	0.025
Glycosaminoglycan degradation	0.54	1.66	0.016

*KEGG, Kyoto encyclopedia of genes and genomes; GSEA, gene set enrichment analysis; ES, enrichment score; NES, normalized enrichment score; NOM p-value, normalized p-value.*

**FIGURE 5:**
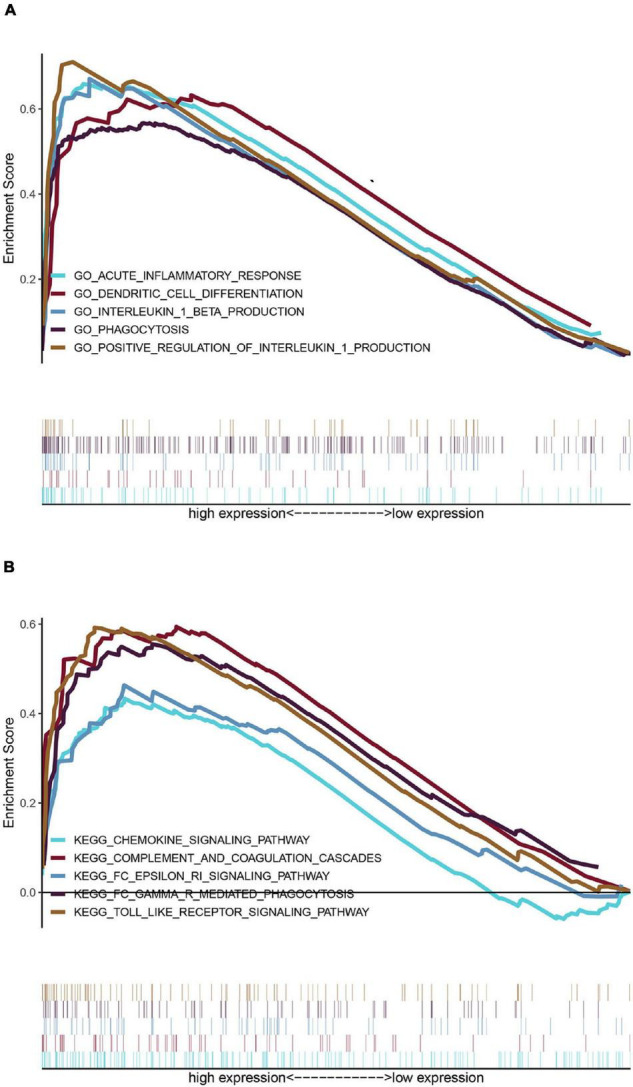
Enrichment analyses *via* gene set enrichment analysis (GSEA). **(A)** Five representative enriched immune-related GO gene sets and **(B)** five representative enriched immune-related KEGG pathways.

The top 20 results of the GO analysis were mainly enriched in immune responses, such as interleukin-1 beta (IL1B) production, phagocytosis, and the regulation of inflammatory response. Five representative immune-related GO terms are visualized in Figure 5A.

The top 20 KEGG pathways were mainly enriched in immune-related pathways [Toll-like receptor (TLR) signaling pathway, NOD-like receptor signaling pathway, *Fc* epsilon *RI* signaling pathway, and Fc gamma R-mediated phagocytosis], immune-related diseases (Leishmania infection and type II diabetes mellitus). Five representative immune-related pathways are shown in Figure 5B.

### PPI Network Construction and Hub Gene Selection

**FIGURE 6:**
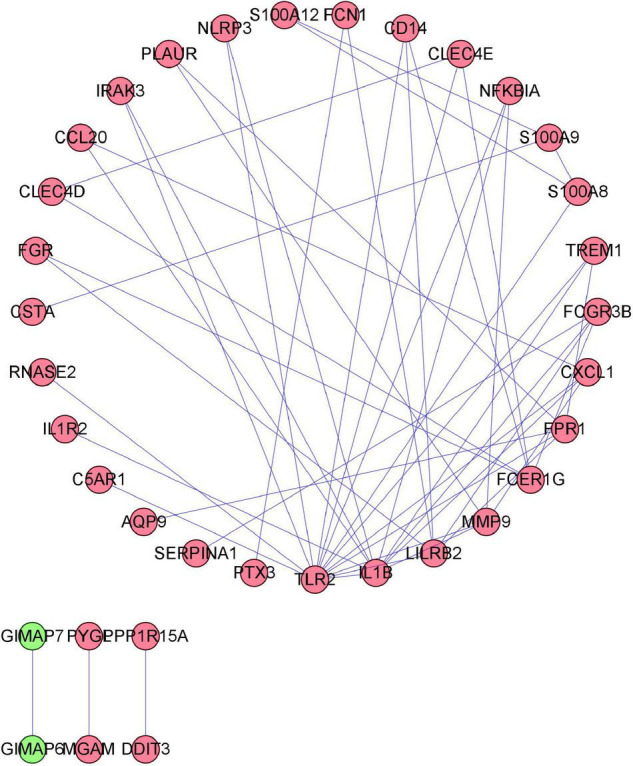
Protein–protein interaction (PPI) network construction. Circles and lines represent genes and the interaction of proteins between genes, respectively. The red represents the upregulated genes. The green represents the downregulated genes.

**FIGURE 7:**
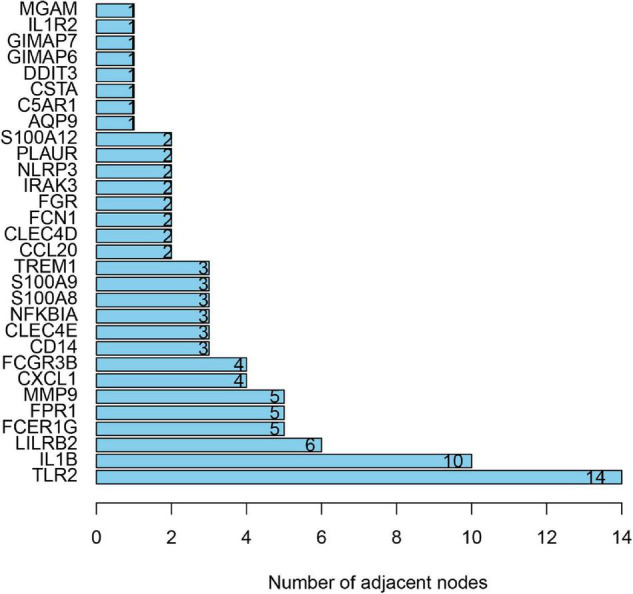
The number of adjacent nodes.

To distinguish the hub genes from the common genes, a DEG PPI network was constructed using the STRING database. As seen in Figure 6, there were 35 nodes and 50 edges in this network. As seen in Figure 7, TLR 2, IL1B, leukocyte immunoglobulin-like receptor subfamily B2 (LILRB2), the Fc fragment of IgE receptor Ig (FCER1G), formyl peptide receptor 1 (FRP1), and matrix metalloproteinase 9 (MMP9) proteins interact with other proteins by >5, which suggested that they were the central nodes of the PPI network. Thus, we chose the top six genes for further research.

### Validation of Diagnostic Values of Hub Genes

**FIGURE 8:**
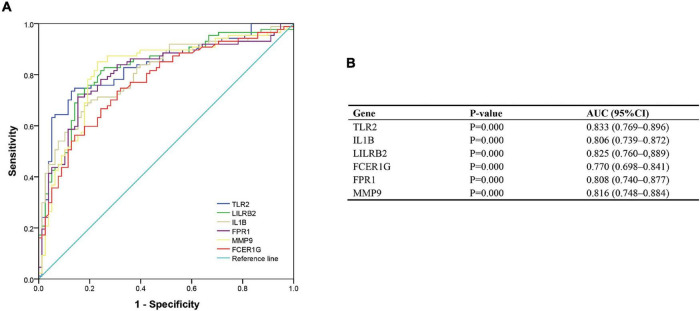
Diagnostic value of top six hub genes with receiver operating characteristic (ROC) curves. **(A)** Analysis with ROC curves. **(B)** Specific value of diagnosis efficiency.

**FIGURE 9:**
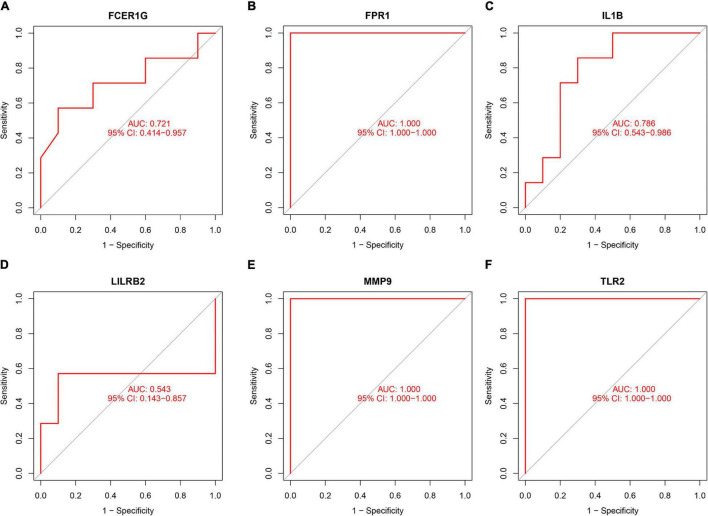
Validation of the diagnostic value of hub genes in the GSE61144 data set. **(A)** Fc fragment of IgE receptor Ig (FCER1G), **(B)** formyl peptide receptor 1 (FPR1), **(C)** interleukin-1B (IL1B), **(D)** leukocyte immunoglobulin-like receptor subfamily B2 (LILRB2), **(E)** matrix metalloproteinase 9 (MMP9), and **(F)** Toll-like receptor 2 (TLR2).

To validate the diagnostic ability of the top six hub genes obtained from the abovementioned analysis, ROC curves were constructed and the AUC value was utilized to determine the diagnostic effectiveness in distinguishing AMI from control sample. Figure 8 shows the AUC for TLR2 was 0.833 (95% CI, 0.769–0.896; *p* = 0.000), AUC for IL1B was 0.806 (95% CI, 0.739–0.872; *p* = 0.000), AUC for LILRB2 was 0.825 (95% CI, 0.760–0,889; *p* = 0.000), FCER1G was 0.770 (95% CI, 0.698–0.841; *p* = 0.000), AUC for FPR1 was 0.808 (95% CI, 0.740–0.877; *p* = 0.000), and AUC for MMP9 was 0.816 (95% CI, 0.748–0.884; *p* = 0.000), indicating that the hub genes had high diagnostic ability. Furthermore, the diagnostic power of hub genes was confirmed in the GSE61144 data set (Figure 9). The results in Figure 9 indicated that the hub genes had high diagnostic ability.

### Immune Cell Infiltration

**FIGURE 10:**
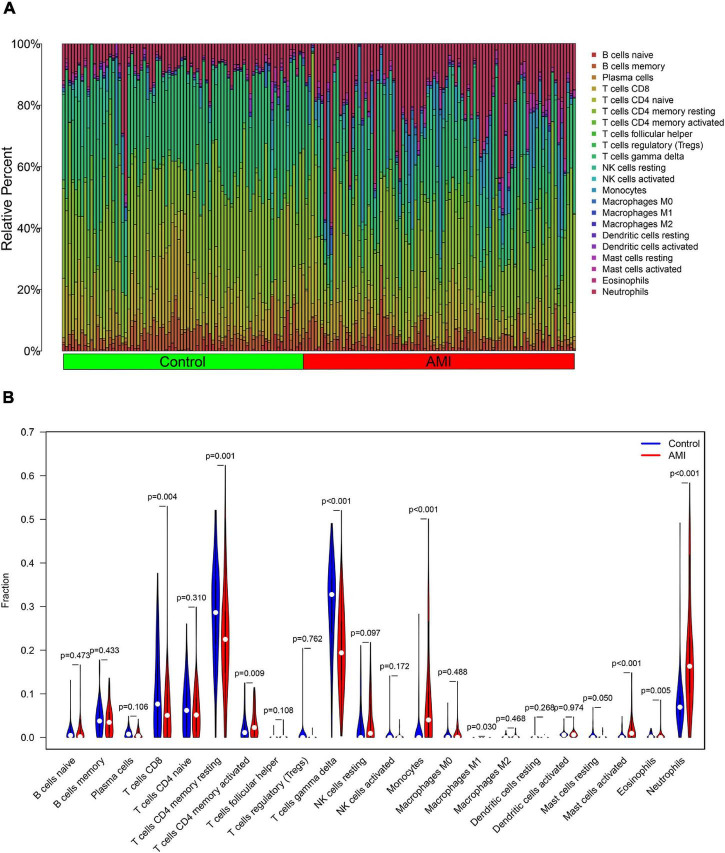
Distribution and visualization of immune cell infiltration. **(A)** The fraction of 22 subsets of immune cells in AMI and control samples. **(B)** The violin graph shows the difference of immune infiltration between AMI and control samples. The control samples are shown in blue and AMI samples are shown in red. The value of value *p* < 0.05.

The composition of immune cells was first explored between AMI tissues and normal control tissues. As shown in Figure 10, the composition of immune cells varied significantly between the different groups. Compared with control group, the AMI group showed a lower fraction of CD8 T cells (*p* = 0.004), CD4 memory resting T cells (*p* = 0.001), gamma delta T cells (*p* < 0.001), M1 macrophages (*p* = 0.030), and eosinophils (*p* = 0.005). However, the AMI group contained a higher proportion of CD4 memory-activated T cells (*p* = 0.009), monocytes (*p* < 0.001), activated mast cells (*p* < 0.001), and neutrophils (*p* < 0.001).

**FIGURE 11:**
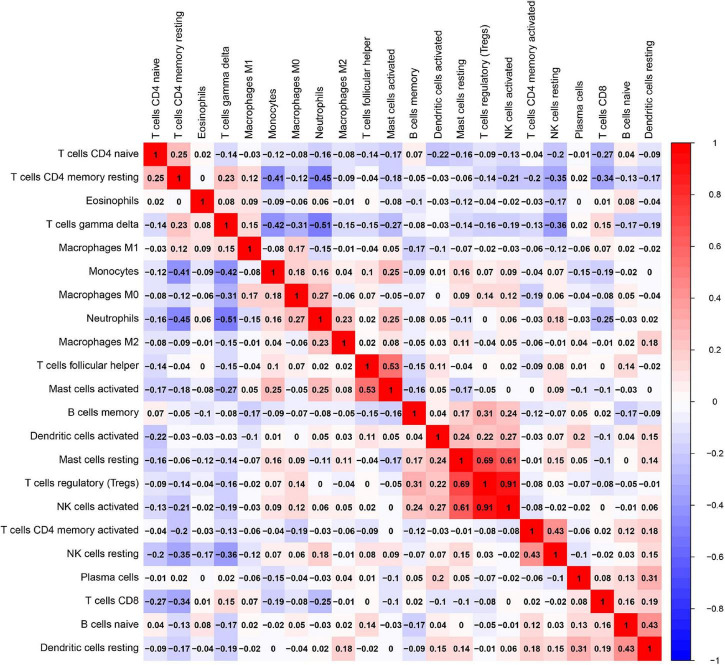
Correlation matrix among fractions of 22 immune cell subtype. Red: positive correlation; white: the same correlation levels; and blue: negative correlation.

Besides, the correlation analysis of 22 types of immune cells was conducted. The score represents the degree of correlation. The results in Figure 11 indicated that activated NK cells and regulatory T cells (Tregs) showed the most synergistic effect. Meanwhile, gamma delta T cells and neutrophils showed the most competitive effect.

### A Correlation Analysis Between the Hub Genes and Infiltrated Immune Cells

**FIGURE 12:**
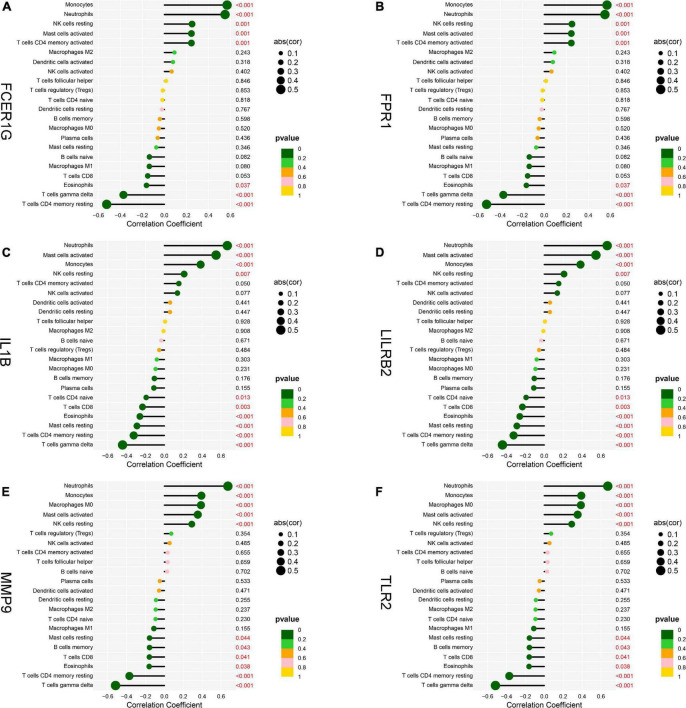
Analysis of correlation between hub genes and immune cells. **(A)** FCER1G, **(B)** FPR1, **(C)** IL1B, **(D)** LILRB2, **(E)** MMP9, and **(F)** TLR2.

To explore the association of the hub genes with the immune cells, we performed the Spearman’s rank correlation analysis. As shown in Figure 12, TLR2 was positively correlated with neutrophils, monocytes, activated mast cells, and resting NK cells and negatively correlated with CD8 T cells, eosinophils, gamma delta T cells, and CD4 memory resting T cells. IL1B was positively correlated with neutrophils, activated mast cells, monocytes, and resting NK cells and negatively correlated with CD4 naïve T cells, CD8 T cells, eosinophils, resting mast cells, CD4 memory resting T cells, and gamma delta T cells. LILRB2 was positively correlated with neutrophils, monocytes, activated mast cells, and resting NK cells and negatively correlated with CD8 T cells, eosinophils, plasma cells, CD4 memory resting T cells, and gamma delta T cells. FCER1G was positively correlated with monocytes, neutrophils, resting NK cells, activated mast cell, and CD4 memory-activated T cells and negatively correlated with eosinophils, gamma delta T cells, and CD4 memory resting T cells. FPR1 was positively correlated with neutrophils, monocytes, activated mast cells, and resting NK cells and negatively correlated with naïve B cells, M1 macrophages, eosinophils, gamma delta T cells, and CD4 memory resting T cells. MMP9 was positively correlated with neutrophils, monocytes, M0 macrophages, activated mast cells, and resting NK cells and negatively correlated with resting mast cells, memory B cells, CD8 T cells, eosinophils, CD4 memory resting T cells, and gamma delta T cells.

## Discussion

Acute myocardial infarction is a highly prevalent disease around world, which remains a leading cause of cardiovascular death. Therefore, timely and appropriate diagnosis of AMI is crucial to improve the prognosis. Previous studies have identified several diagnostic biomarkers for AMI including cardiac troponins T (cTnT) and I (cTnI), CK-MB (22). Cardiac troponins T (cTnT) has considered as the gold standard in the diagnosis of AMI, while its specificity is limited (23). Furthermore, recent research found that immune cell infiltration is closely related to the occurrence and development of AMI (24). Hence, we aimed to identify novel diagnostic biomarkers to screen for patients with AMI and reveal the role of immune cells in AMI.

In this study, we collected three data sets from the GEO database, and a total of 71 DEGs, including 68 upregulated genes and 3 downregulated genes, were found. Functional enrichment analyses were also performed, and the results demonstrated that DEGs were mainly enriched in inflammatory and immune-related functions. BP terms of GO were mainly enriched in immune responses, such as neutrophil activation, neutrophil-mediated immunity, and neutrophil migration. Through the KEGG pathway analysis, we found DEGs mainly enriched in immune system diseases and immune-related pathways, such as TB, IL-17 signaling pathways, NF-kappa B signaling pathway, and TNF signaling pathway. Moreover, the functional enrichment analysis based on DEGs between AMI and control tissues showed the results similar to those of GSEA. These findings suggested that immune and inflammatory systems are related to the pathogenesis of AMI (25). Interleukin-17 signaling pathway participates in the immune response, the production of inflammatory mediators, and poor remodeling after AMI (26). Moreover, NF-kappa B signaling pathway contributes to the enhanced inflammatory response in unstable angina, and NF-kappa B was considered to be involved in plaque destabilization (27). The IL-17 family has the capacity to activate the NF-kappa B signaling pathway and ultimately causes the expression inflammatory cytokines (28). The TNF signaling has been confirmed to be involved in the inflammatory cell accumulation and the cardiac remodeling following AMI (29). These studies confirmed that the immune response plays an important role in AMI. Thus, the exploration of novel biomarkers of AMI associated with immune cell infiltration *via* a bioinformatic analysis shows promise for the treatment of AMI.

Utilizing the STRING database, we constructed the PPI network and identified the hub genes. The top six hub genes, TLR2, IL1B, LILRB2, FCER1G, FPR1, and MMP9, were filtered for further study. TLRs contribute to AMI pathogenesis (30). TLR2 and TLR4 agonists trigger platelet activation in patients with AMI through NF-kappa B. Previous studies showed that the activation of TLR2 and TLR4 induced the innate immune response, increasing infarction size and influencing ventricular remodeling (31, 32). Another research indicated that the deficiency of TLR2 can alleviate inflammatory response and prevent cardiac remodeling after AMI (33).

Interleukin-1 beta is the best characterized cytokine of the IL-1 receptor family, which has been reported to serve as a key mediator of the inflammatory and immune response (34). IL1B blockage shows a promise for the treatment of AMI because of its central role in the regulation of an inflammatory response that results from AMI (35). LILRB members are the immunomodulatory receptors that can inhibit immune cell activation *via* cytoplasmic immunoreceptor tyrosine-based inhibitory motifs (ITIM) (36). LILRB2 has been reported to suppress the neutrophil overactivation and alleviate excessive inflammatory response (37). Yan et al. analyzed the expression of B cell-associated genes among patients with AMI, stable angina and health controls, a finding that LILRB2 and LILRB3 levels were statistically increased in AMI (38). The FCER1G gene is located on 1q23.3 (39). FCER1G is an innate immune gene and shows a negative modulatory role in the B-cell response (40). Formyl peptide receptors (FPRs) are G protein-coupled receptors that are involved in multiple pathologies (41). FPR1 knock-down mice showed decreased infarction size, suppressed inflammatory response, and cardiomyocyte apoptosis (42). More recent research suggested that FPR1 could be a novel biomarker for the diagnosis and the treatment of AMI (43, 44). Matrix metalloproteinases (MMPs) are a major family of zinc-dependent proteases that degrade diverse proteins in an extracellular matrix, which has obtained more and more attention in recent years. Recent studies have found that MMPs are closely related to immune cells and the ablation of MMP9 decreases the infarction size in AMI (45, 46). Wei et al. found that trimetazidine inhibited MMP2 and MMP9 expression and prevent cardiac rupture in mice with AMI (47). Thus, the evidence suggest that hub genes are involved in the development of AMI, which may be act as the potential therapeutic targets for AMI.

Then, we conducted the analysis of immune infiltration of AMI through the CIBERSORT algorithm, and analyzed the correlation between hub genes and infiltrating immune cells. The results from CIBERSORT showed that multiple immune cell subtypes were closely correlated with the important BPs of AMI. A higher fraction of CD4 memory-activated T cells, monocytes, activated mast cells and neutrophils, and a decreased fraction of CD8 T cells, CD4 memory resting T cells, gamma delta T cells, M1 macrophages, and eosinophils. Furthermore, the results of the correlation analysis between TLR2, IL1B, LILRB2, FCER1G, FPR1, and MMP9, and immune cells found that TLR2, IL1B, LILRB2, FCER1G, FPR1, and MMP9 all correlated with neutrophils, monocytes, resting NK cells, gamma delta T cells, and CD4 memory resting T cells. In recent years, there is a growing recognition that innate immune system plays an important contribution to the progression of heart disease (48). After AMI, a variety of immune cells, including neutrophils and monocytes, are recruited for the heart and trigger an intense inflammatory response (49). Neutrophils mediate the damage of infarcted cardiomyocytes *via* releasing matrix-degrading enzymes, and are also involved in postinfarction remodeling (50). Thus, these hub genes associated with immune cells could serve as potential therapeutic targets for AMI treatment. Moreover, our results (Figure 10) showed a synergistic effect between activated NK cells and Tregs cells as well as a competitive effect between gamma delta T cells and neutrophils. A few studies have reported the relationship. Accordingly, we speculate that activated NK cells and Tregs mutually reinforce their effects as well as gamma delta T cells and neutrophils mutually antagonize the activities of each other during AMI. However, the correlation between these immune cells needs to be further studied.

The limitations of our study should be considered. First, the number of original cases in each data set was low, thus we selected three data sets. Second, as the study was retrospective, the corresponding clinical information was lacking. Finally, this study was conducted *via* the bioinformatics analysis, further prospective studies are needed to determine the results *in vivo*.

## Conclusion

In conclusion, TLR2, IL1B, LILRB2, FCER1G, FPR1, and MMP9 were identified as novel diagnostic biomarkers of AMI and their diagnostic values were verified using SPSS data analysis. Moreover, immune cells, such as neutrophils, monocytes, resting NK cells, gamma delta T cells, and CD4 memory resting T cells, may be involved in the occurrence and development of AMI. These immune cells may provide a novel perspective on immunotherapeutic targets for patients with AMI.

## Data Availability Statement

Publicly available datasets were analyzed in this study. This data can be found here: All the raw data used in this study are available in the public GEO database (https://www.ncbi.nlm.nih.gov/geo/; Accession numbers: GSE66360, GSE48060, GSE60993, and GSE61144).

## Author Contributions

YW and WX conceived and designed this study. TJ, JH, ZX, HC, LL, and JP collected the data as well as provided assistance in data analysis. TJ, JH, and WX provided significant suggestions on the methodology. YW conducted data management and bioinformatics analysis and drafted the manuscript. YW, TJ, JH, ZX, HC, LL, and JP edited and revised the manuscript. All authors read and approved the final manuscript.

## Conflict of Interest

The authors declare that the research was conducted in the absence of any commercial or financial relationships that could be construed as a potential conflict of interest.

## Publisher’s Note

All claims expressed in this article are solely those of the authors and do not necessarily represent those of their affiliated organizations, or those of the publisher, the editors and the reviewers. Any product that may be evaluated in this article, or claim that may be made by its manufacturer, is not guaranteed or endorsed by the publisher.

## Footnotes

1
http://www.ncbi.nlm.nih.gov/geo/
